# Supplementary material for: Patterns of Intron Gain and Loss in Fungi
Source: PLoS Biol. 2004 Nov 30;2(12):e422. doi: 10.1371/journal.pbio.0020422 (PMC532390; doi:10.1371/journal.pbio.0020422)
Supplement: Table S1 — Also available at http://genes.mit.edu/NielsenEtAl/. (4.3 MB ZIP). [file pbio.0020422.st001.zip › NielsenEtAl/html/185.html]

AN2495.1.NCU00569.1.MG03071.1.FG08552.1


```
 CLUSTAL W (1.82) Multiple Sequence Alignments - Introns Inserted


Sequence 1: NCU00569.1	134 aa
Sequence 2: FG08552.1	128 aa
Sequence 3: MG03071.1	132 aa
Sequence 4: AN2495.1	158 aa
Alignment Length: 171 aa
Number Identitical Residues: 49 aa
Alignment Score (without introns) 2558


MG03071.1 	MSDIVPPTSATTSDPPPAPSSTADEPKYGGHTRFEIELE~FVQALGNPIYLNHLAVN--K
NCU00569.1	MASDPMSVNSSAREPPAPPGSGADEPKYGGYTRFELELE0FVQALGNPLYLNHLASR--K
FG08552.1 	MAEETPQDVSMESPPPAPMQTDQ-EPKYGGYSRFEIELE0FVQSLANPAYLNHLASQ--K
AN2495.1  	MAQP----QNDQAQPPPATLTNP---------RFTLELE~FVSSLANPYYLSHLAVNYPS
          	*:.           **..  :           ** :*** **.:*.** **.*** . ..

MG03071.1 	VL---------------SQP---AFVAYLDYLQ-YWTRPPYVKYLTYPGPTLRSLKLLQQ
NCU00569.1	LL---------------SNP---AFIAYLDYLQ-YWSRPPYLKYITYPGPTLKNLELLQQ
FG08552.1 	LL---------------SQP---AFVAYLAYLR-YWSKPPYLKYLTYPGPTLRHLELLQQ
AN2495.1  	LLGISKSGNENEVNDDNSDPDAEAFAAYLAYLYSYWKTPEYSQFLTHPGATLRALRLLQE
          	:*. :.:..... ....*:*.:.** *** ** :**. * * :::*:**.**: *.***:

MG03071.1 	EKFRQDIIMPELVNALV---EQGMRAAVEWHMEDKKP---------------
NCU00569.1	EKFRQDIISPDLVERLR---LEGMKAGIDWHRESFPSTA-------------
FG08552.1 	ETFRQQIISPDVVRALM---EEGMKASVDWHREN------------------
AN2495.1  	EKFRRDIIRPQVIERLAGVDLNGEQSAAEAGEQNTEQNKGDRGNVENQHGKT
          	*.**::** *:::. * . . :* ::. :   :.   . .. .. ... ..:
```
